# Cognitive medicine – a new approach in health care science

**DOI:** 10.1186/s12888-018-1615-0

**Published:** 2018-02-08

**Authors:** Anders Wallin, Petronella Kettunen, Per M. Johansson, Ingibjörg H. Jonsdottir, Christer Nilsson, Michael Nilsson, Marie Eckerström, Arto Nordlund, Lars Nyberg, Katharina S. Sunnerhagen, Johan Svensson, Beata Terzis, Lars-Olof Wahlund, H. Georg Kuhn

**Affiliations:** 1 University of Gothenburg, Sahlgrenska Academy, Institute of Neuroscience and Physiology, Gothenburg, Sweden; 2 Nuffield Department of Clinical Neurosciences, University of Oxford, Oxford, UK; 3 Institute of Medicine, Sahlgrenska Academy at University of Gothenburg, Gothenburg, Sweden; 4 Institute for Stress Medicine, Region Västra Götaland, Gothenburg, Sweden; 5 Department of Food, Nutrition and Sport Science, University of Gothenburg, Gothenburg, Sweden; 6 Department of Clinical Sciences, Clinical Memory Research Unit, Lund University, Lund, Sweden; 7 University of Newcastle, Hunter Medical Research Institute, Newcastle, NSW Australia; 8 Center for Functional Brain Imaging and Department of Radiation Sciences & Integrative Medical Biology, Umeå University, Umeå, Sweden; 9 Department of Endocrinology, Skaraborg Central Hospital, Skövde, Sweden; 10 Frösunda Omsorg AB, Solna, Sweden; 11 Department of Neurobiology, Care Sciences and Society (NVS), Karolinska Institute, Solna, Sweden; 12 Department of Neurology, Center for Stroke Research, Charité – Universitätsmedizin, Berlin, Germany

**Keywords:** Neurocognitive disorders, Classification of diseases, Disability, Rehabilitation, Mental functions, Learning and memory, Executive control, Societal challenge, Stress and environment

## Abstract

**Background:**

The challenges of today’s society call for more knowledge about how to maintain all aspects of cognitive health, such as speed/attention, memory/learning, visuospatial ability, language, executive capacity and social cognition during the life course.

**Main text:**

Medical advances have improved treatments of numerous diseases, but the cognitive implications have not been sufficiently addressed. Disability induced by cognitive dysfunction is also a major issue in groups of patients not suffering from Alzheimer’s disease or related disorders. Recent studies indicate that several negative lifestyle factors can contribute to the development of cognitive impairment, but intervention and prevention strategies have not been implemented. Disability due to cognitive failure among the workforce has become a major challenge. Globally, the changing aging pyramid results in increased prevalence of cognitive disorders, and the diversity of cultures influences the expression, manifestation and consequences of cognitive dysfunction.

**Conclusions:**

Major tasks in the field of cognitive medicine are basic neuroscience research to uncover diverse disease mechanisms, determinations of the prevalence of cognitive dysfunction, health-economical evaluations, and intervention studies. Raising awareness for cognitive medicine as a clinical topic would also highlight the importance of specialized health care units for an integrative approach to the treatment of cognitive dysfunctions.

## Background

### Cognitive medicine – an emerging field

Cognition is a summary term for thought processes used in interactions with other humans and the environment and is often described as a system of cognitive functions ranging from visual perception to social cognition [1]. A longer work life and the need for skill development due to rapid technological development and global competition have led to increased demands on the individual to sustain a high cognitive performance. Medical advances have been rapid, but the cognitive implications of various diseases have not been sufficiently addressed. Furthermore, a growing number of elderly combined with changed lifestyle have resulted in many health-related consequences, including disability due to cognitive impairment. Thus, a major challenge for today’s society is to maintain cognitive health in order to avoid disability.

**Fig. 1:**
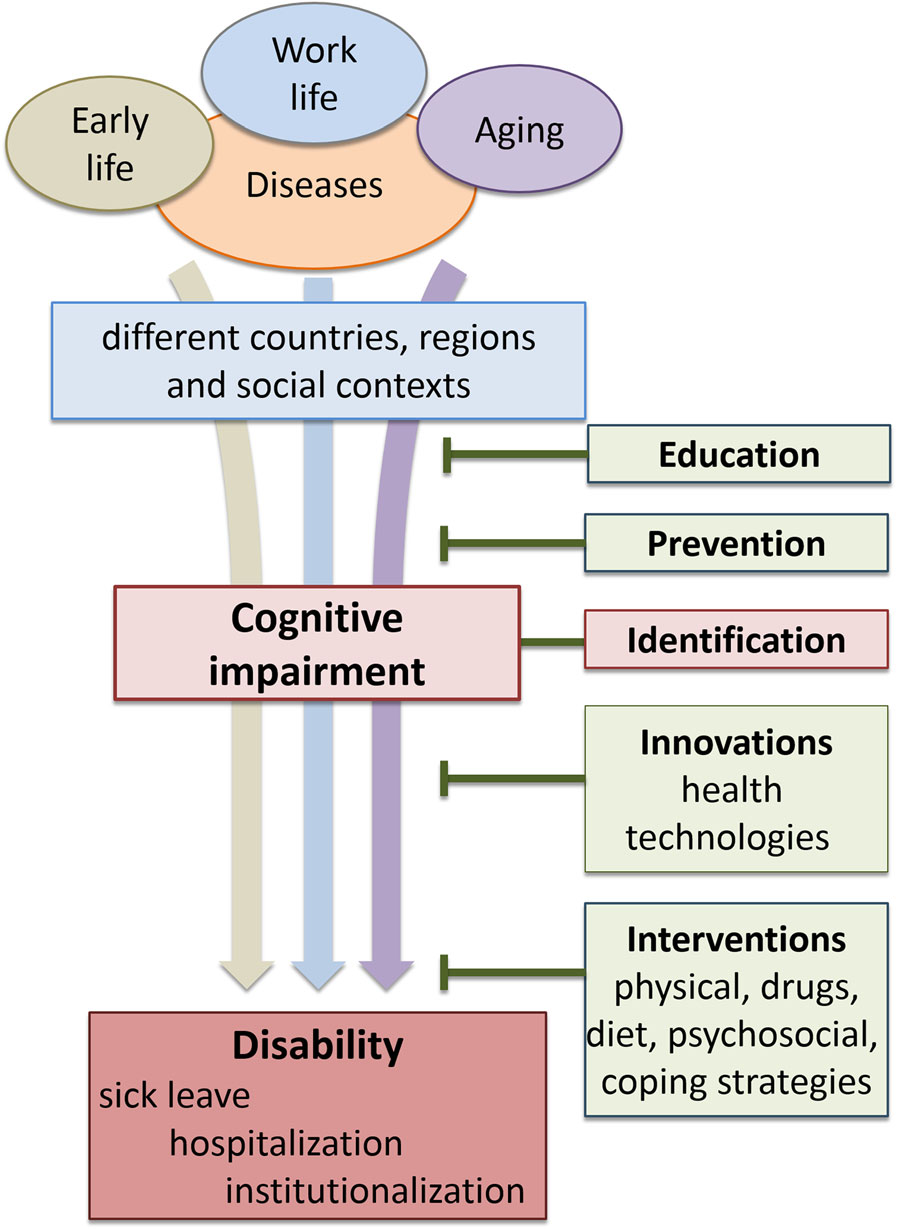
Cognitive medicine is an emerging topic dealing with cognitive impairment during the life course in medical and associated contexts

Cognitive impairment or decline is part of the essential symptomatology in a wide spectrum of medical conditions over the life course, including diseases that are not primarily associated with cognitive deficits, such as heart insufficiency, infections and cancer. In this paper, we argue for the need to develop the concept of cognitive medicine as an umbrella term covering aspects of cognition across different health conditions and diseases, to merge and increase knowledge of how to prevent, diagnose, cope and intervene with cognitive decline (Fig. 1). We define cognitive medicine as a field of knowledge and a research area focused on the identification, prevention, study and treatment of cognitive impairment and decline.

Major research tasks in cognitive medicine are to uncover cellular and molecular mechanisms underlying decline of cognitive functions, to determine the prevalence and incidence of cognitive dysfunctions in epidemiological populations and patient populations, health-economical evaluations of cognitive impairments, and intervention studies to investigate the effects of medical treatments and lifestyle interventions. Furthermore, new tools need to be developed such as high- and low-tech diagnostic procedures for cognitive profiling, imaging, biochemistry and genetics. The knowledge from cognitive medicine will also be useful to clarify the role of the current memory clinics, to form health care units for patients with diseases associated with cognitive decline and to implement education programs for a medical specialty in cognitive medicine.

## Main text

### Cognitive functions throughout the life course

The cognitive system is usually believed to comprise six key functions, which in turn are divided in subdomains [1]. The key cognitive functions are perceptual-motor function (including visuospatial function), attention, learning and memory, language, executive function and social cognition. There is no clear-cut border between cognitive, emotional and volitional functions as cognition comprises a volitional component and influences emotional reactions and decision-making. When the information process takes place, it engages the cognitive functions in a specific order. Information obtained by the sensory systems is selected for further processing with the aid of attention. The selected information can then be retained over shorter time periods by a working-memory system. Broadly defined, executive functions include initiation, motivation, planning, and control of goal-oriented basic and complex actions, and incorporate more basic executive processes such as shifting, updating, and inhibition. Various long-term memory systems allow information to be retained over weeks, months, and even years. Memory systems are specialized for different types of information (facts, events and procedures). Semantic memory, the long-term memory for facts, is closely related to language functions. Language, i.e. symbolic representation according to set rules, and visuospatial functions, i.e. comprehending and conceptualizing visual representations and spatial relationships, are used in certain contexts. Social cognition is a set of complex cognitive processes centered on social interactions, such as understanding other persons’ wishes and needs. Related to social cognition is metacognition, which refers to the ability to reflect upon one’s own cognitive processes.

Most cognitive capacities develop from infancy through childhood and adolescence into adulthood and reflect the maturation of the central nervous system over time. By 10 years of age, when the brain has begun to reach its adult size, children have already undergone substantial development in cognitive functions [2, 3]. Cognition continues to develop in parallel with a refinement of neural networks via synaptic strengthening and pruning as well as myelination. In aging, many but not all cognitive functions begin to decline. Examples of cognitive functions that are well-preserved into older age include memory for facts, social cognition, and more general functions that tap into well-consolidated information [4]. Age-sensitive cognitive functions include working memory, certain forms of attentional functions, episodic long-term memory, and several executive functions. The normal variation in cognitive capacity between individuals increases with age, and while some older adults show marked cognitive decline other maintain high levels of cognitive functioning [5].

### Disability

An independent and productive life requires that cognitive functions are preserved or compensated for, otherwise disability will arise. The World Health Organization (WHO) has defined *disability* as an umbrella term covering impairments, activity limitations, and restrictions in participation [6]. An impairment is a problem in body function or structure; an activity limitation is a difficulty encountered by an individual in executing a task or action; while a participation restriction is a problem experienced by an individual in involvement in life situations. Therefore, disability cannot be seen as just a health problem but as a phenomenon, which is reflecting the interaction between features of a person’s body and features of the society in which he or she lives. In the “World Report of Disability “from WHO and the World Bank it is shown that not only are persons with disability at an economic disadvantage caused by difficulties in getting/keeping a job. There is also the burden on the family, where family members often have to stay at home to take care of the disabled person [7].

Since impairment of cognitive functions always has an impact on various types of human activities, *cognitive impairments are unambiguously linked to disability.* The consequences of the impaired cognition depend on the activities required as well as the environment these will occur in and both relate to the societal circumstances a person may encounter. Examples of activities that can be affected include planning daily actions, accomplishing tasks in working life, handling stress, managing personal economic matters, communicating with others and participating in leisure activities. For example, both stroke and schizophrenia conditions might affect cognition but the consequences can be different depending on other impairments as well as environmental and personal factors [8–10]. Here, the continuing development of tools, as well as restorative and compensatory measures are of great importance.

### Basic research perspective

There are several disorders that give rise to cognitive dysfunctions such as early-life neuropsychiatric disorders, trauma, epilepsy, Alzheimer’s disease, Parkinson’s disease and other neurological disorders, bipolar disorders, substance abuse and other psychiatric disorders, infectious and inflammatory diseases, stroke and other vascular disorders, cancer and endocrine diseases. The idea of cognitive medicine is to integrate research on behavioral, cellular and molecular mechanisms associated with cognitive dysfunctions in divers disorders.

Although Alzheimer’s disease is the most studied cognitive disorder, the key pathobiological events in the early phases of the disease have not yet been clearly defined. There are indications that the onset of the pathological events in Alzheimer’s disease might occur already two decades before cognitive impairment is debilitating [11]. Therefore, early symptoms of Alzheimer’s disease could coincide with work life-related cognitive alterations and psychiatric conditions such as anxiety, depression and stress-related exhaustion. Furthermore, an important focus of cognitive medicine is to investigate whether cognitive disorders late in life are associated with, or even casually linked to, lifestyle-related diseases earlier in life such as hypertension, diabetes, and obesity.

There is overlap in pathology between Alzheimer’s disease and other cognitive disorders such as vascular cognitive impairment [12, 13], and the affected pathways can be detected using cognitive profiling. Alzheimer’s disease, also early in the course of the disease, is characterized by memory loss and difficulties in interpreting sensory information. In contrast, subcortical small vessel disease displays a cognitive pattern of early dysfunction of mental speed and executive capacity [14, 15]. Cognitive assessment can be used to distinguish Alzheimer’s disease from other dementing disorders such as subcortical small vessel disease and also to evaluate to what extent there is co-existence of the conditions.

### Prevalence of cognitive dysfunction and health economy

WHO estimated that 47 million people have advanced cognitive impairment worldwide, and the estimation is that this will increase to near 75 million in 2030 and to 132 million in 2050. According to estimations by the Alzheimer’s Association, 15-20% of individuals aged 65 and above suffers from mild cognitive impairment. However, different studies have shown highly variable results depending on the cohort studied and the cognitive tests used. A major task for researchers working in the field of cognitive medicine is therefore to determine, in population-based as well as patient cohort studies, the prevalence of cognitive functions in work life, education, rehabilitation, and chronic diseases. For this purpose, the development of new, standardized tools to measure cognitive functions in large cohorts is needed. Based on the prevalence and incidence of cognitive dysfunctions and the degrees of disability, health economic calculations can be performed to determine focus area of specific interest for cognitive medicine and to facilitate health care planning.

### Working life perspective

Cognitive dysfunctions during work life is one focus area in cognitive medicine. In today’s society, the rapid development in information technology, social media, and transmission of information continually changes life conditions. Adaptation to the changing cognitive demands is crucial and cognitive flexibility, which is a component of executive function, has become ever more important. Reduced working performance due to a mismatch between the cognitive demands and cognitive function is a growing challenge for many workplaces. We are just beginning to understand the cognitive impairments that are associated with work-related stress and how to prevent and treat these deficiencies. Moreover, medical professionals from occupational and insurance medicine among others are in need of novel tools for evaluating the relationship between cognitive functions, working capacity and medical symptoms for the assessment of cognitive impairments in different patient groups, objectives that teams of cognitive medicine specialists could accomplish.

### Global perspective

The rapid increase in the prevalence of advanced cognitive impairment is a global issue since the number of elderly individuals increases also in developing countries with rapid population growth, which will result in a marked rise in the prevalence of age-related brain diseases [16]. In addition, environmental aspects have attracted a higher level of attention as life-long exposure to infectious agents and environmental toxins may increase the risk for cognitive impairment [17, 18]. In developing countries, urbanization and increased psychosocial demands in working life will not only influence cognitive health in the elderly but also in young and middle-aged people. Moreover, a major challenge from a medical perspective is the contextual aspect of cognitive performance. This means that the diversity of cultures worldwide influences the expression, manifestation and consequences of cognitive dysfunction, implying that a socio-cultural approach, besides medical and psychological advances, is needed.

### Prevention and intervention

There are at present few effective medical treatments for cognitive disorders. Obviously, randomized clinical trials for new treatments affecting the underlying pathological mechanisms are needed. Another aspect is that negative lifestyle factors contribute substantially to the development of cognitive impairments [19], but successful lifestyle-related prevention and intervention strategies have yet to be implemented. In our aging population with increased stress-related disorders and sedentary lifestyle, cognitive impairments as a cause of disability has risen to epidemic proportions globally. A major question that has to be addressed by cognitive medicine is whether cognitive decline, seemingly due to preventable lifestyle factors, is partly or fully reversible if adequate treatment is given.

A sedentary lifestyle is not only of importance during adulthood or aging. There is clear evidence that lack of exercise and obesity correlate with lower cognitive performance both at school age in school, as well as later in life [20–22]. Large longitudinal cohort studies have indicated increased risk for psychiatric diseases, such as depression and anxiety disorders, and brain diseases, such as stroke and major neurocognitive disorders, later in life in subjects with low physical performance during late adolescence [23–25]. In light of the epidemic development of obesity and physical inactivity worldwide, research in cognitive medicine will also address how to design successful early prevention and interventions in children and adults in order to develop and maintain an active lifestyle thereby increasing resilience against cognitive dysfunctions throughout the life course.

### Integration with existing medical care practice

Memory clinics or similar health care units deal mainly with Alzheimer’s disease and related neurocognitive disorders. The responsibilities of such units may be expanded to also include investigation of various disorders usually not classified as classical memory disorders, but which may exhibit cognitive dysfunctions as essential component that worsen rehabilitation and prevent return to work. The overall aim of such health care units, in addition its traditional task, would be to identify and evaluate severity, profile and consequences of cognitive impairments for treatment of the somatic and psychiatric disorders. Furthermore, competence in cognitive medicine can be increased by introducing 6-12 months practice at these units for doctors trained in neurology, psychiatry, rehabilitation medicine or general practice. Nurses and psychologists can increase their competence in cognitive medicine in a similar way.

## Conclusions

Cognitive medicine is aiming at creating specific knowledge about the relationships between disease processes, cognitive functions and disabilities. There is a need to establish new tools for identification of cognitive impairments in various medical conditions. Without such cognitive assessment instruments taking into account the whole spectrum of cognitive functions, it will be difficult to interpret genetic, biochemical, and imaging data both in research and clinical practice. In addition, the highly technological approach using fluid and imaging biomarkers is not available in all contexts and countries, which means that precise and standardized cognitive assessment may be the only way of getting information about the suspected cognitive disorder. Based on our argumentation, we recommend that cognitive medicine becomes a recognizable field in healthcare, education and research programs.

## Abbreviations

DSM-5: The Diagnostic and Statistical Manual of Mental Disorders, Fifth Edition
WHO: The World Health Organization
